# Supplementary material for: MDL-001: An Oral, Direct-Acting Broad-Spectrum Antiviral for Influenza-like Illness (ILI) and Chronic Hepatitis In Vivo
Source: Pharmaceuticals (Basel). 2026 Oct 18;19(10):1639. doi: 10.3390/ph19101639 (PMC13708324; doi:10.3390/ph19101639)
Supplement: Supplementary file 1 [file pharmaceuticals-19-01639-s001.zip › pharmaceuticals-4605343-supplementary.pdf]

Supplementary Tables

Supplementary Table S1. In vitro antiviral activity of MDL-001 by virus family, virus, and assay system. *Bold rows appear in Figure 1.*

	Strain	Cell Line	EC_{50} (µM)	CC_{50} (µM)	SI_{50}	Assay Type	Serum/protein condition:	Free Fraction EC_{50} (nM)	Free Fraction EC_{90} (nM)
Orthomyxoviridae									
**Influenza A**	**H1N1/California/04/09 (WT)**	**MDCK**	**0.165**	**15.1**	**91.2**	**Plaque (PFU)**	**0.3% BSA**	**41.3**	**47.3**
	**H3N2/Victoria/361/2011**	**MDCK**	**0.498**	**15.1**	**30.2**	**Plaque (PFU)**	**0.3% BSA**	**125**	**194**
**Influenza B**	**Victoria/Florida**	**MDCK**	**0.554**	**15.1**	**27.1**	**Plaque (PFU)**	**0.3% BSA**	**139**	**256**
	**Yamagata/Brisbane**	**MDCK**	**0.625**	**15.1**	**24.1**	**Plaque (PFU)**	**0.3% BSA**	**156**	**297**
Pneumoviridae									
**RSV**	**A2**	**A549**	**1.59**	**7.2**	**4.5**	**VYR (luciferase reporter)**	**10% FBS**	**79.4**	**127**
Coronaviridae									
**SARS-CoV-2**	**USA-WA1/2020**	**HeLa-ACE2**	**0.75**	**>50**	**>66.7**	**IF**	**2% FBS**	**188**	**345**
	**Alpha (B.1.1.7)**	**HeLa-ACE2**	**0.83**	**>50**	**>60.2**	**IF**	**2% FBS**	**208**	**505**
	**Beta (B.1.351)**	**HeLa-ACE2**	**1.05**	**>50**	**>47.6**	**IF**	**2% FBS**	**263**	**595**
	**Delta (B.1.617.2)**	**HeLa-ACE2**	**0.74**	**>50**	**>67.6**	**IF**	**2% FBS**	**185**	**423**
	**Omicron (B.1.1.529)**	**HeLa-ACE2**	**0.75**	**>50**	**>66.7**	**IF**	**2% FBS**	**188**	**345**
	Mouse Adapted-WA1	HeLa-ACE2	0.712	7.652	10.7	IF	2% FBS	178	300
**α-coronavirus**	**229E**	**MRC-5**	**0.11**	**1.03**	**9.4**	**CPE**	**2% FBS**	**27.5**	**47.5**
	229E	Huh7	0.42	8.4	20.0	CPE	2% FBS	105	NC
**β-coronavirus**	**OC43**	**A549-ACE2**	**0.49**	**>15**	**>30.6**	**CPE**	**2% FBS**	**123**	**330**
Flaviviridae									
**Hepatitis C**	**Genotype 1b (Con1)**	**Huh-luc/neo-ET**	**3.28**	**>10**	**>3.0**	**Replicon**	**10% FBS**	**164**	**460**
	**Genotype 2a (JFH-1)**	**Huh7.5.1**	**1.54**	**>10**	**>6.5**	**Replicon**	**10% FBS**	**77.0**	**300**
Hepadnaviridae									
**Hepatitis B**	**HepAD38**	**AD38**	**1.04**	**4.5**	**4.3**	**qPCR**	**10% FBS**	**51.8**	**87.5**
Kolmioviridae									
**Hepatitis D**	**HDV-1 (genotype I) HBV Co-infection**	**hNTCP-Huh7**	**2.13**	**>5**	**>2.3**	**Antigen ELISA**	**10% FBS**	**106**	**152**

CPE: Cytopathic Effect; VYR: Viral Yield Reduction; IF: Immunofluorescence; NC: not calculated.

Supplementary Table S2. Lung log10 viral reduction from vehicle (Influenza Study).

**Lung Burden (Day 5 p.i.)**	**Vehicle**	**Oseltamivir (TAM) (clinical eq. dose)**	**MDL-001**
**Log_{10} Reduction (mean)**	—	2.7	2.6
**95% CI (Log_{10} Reduction)**	—	(2.6, 2.9) ****	(2.3, 2.8) ****
**RNA copies/mg (geometric mean)**	3.2×10^6	5.9×10^3	8.9×10^3
**95% CI (RNA copies/mg)**	$(2.4 \times 10^6, 4.4 \times 10^6)$	$(5.5 \times 10^3, 6.3 \times 10^3)$	$(4.8 \times 10^3, 1.6 \times 10^4)$
	**Superiority Comparison**	**Non-Superior (*P* = 0.14)**	
	**Equivalency Comparison Difference 90% CI (MDL-001 - TAM)**	**Equivalent (−0.3 to 0.0 log_{10})**	

*Geometric means and 95% confidence intervals are shown. (n=3/ group). Significance versus vehicle was tested by two-way ANOVA (****P < 0.0001). The MDL-001 versus clinical equivalent mouse dose of oseltamivir active-comparator superiority comparison was performed on animal-level absolute lung viral RNA reductions by two-sided unpaired Welch's unequal-variance t-test (P = 0.14). Equivalence was assessed by two one-sided tests (TOSTs) against a pre-specified margin of ±0.5 log10. See Methods.*

Supplementary Table S3. Lung log_{10} viral reduction from vehicle (SARS-CoV-2 Study).

**Lung Burden**	**Vehicle**	**MDL-001**	**Nirmatrelvir** *Owen et al.* (16) D4; $CCID_{50}$; n = 12	**Molnupiravir** *Jeong et al.* (17) D5; $TCID_{50}$; n = 5
**Log_{10} Reduction** (mean)	—	2.9<br>3 dp.i.	1.4<br>4 d.p.i, $CCID_{50}$	1.0<br>5d.p.i, $TCID_{50}$
**95% CI** (Log_{10} Reduction)	—	(1.5, 4.3)<br>***	SD = 0.648, n = 12	SD = 0.508, n = 5
**$TCID_{50}$/mL** (geometric mean)	3.4×10^7	4.1×10^4	**Comparison:**	
**95% CI** ($TCID_{50}$/mL)	(4.2×10^6, 2.7×10^8)	(2.4×10^3, 7.0×10^5)	**MDL-001 Superior**<br>($P = 0.023$)	**MDL-001 Superior**<br>($P = 0.008$)

*Geometric means and 95% confidence intervals are shown for the MDL-001 study (n=9/group). Significance versus vehicle was tested by two-way ANOVA (***$P < 0.001$). Nirmatrelvir and molnupiravir values are literature-comparator statistics extracted from Owen et al. and Jeong et al. Nirmatrelvir was measured at Day 4 p.i. by $CCID_{50}$, molnupiravir was measured at Day 5 p.i. by infectious-titer assay, and MDL-001 was measured at Day 3 p.i. by $TCID_{50}$. SD values in the literature columns are the variance inputs used for the Welch comparisons. Literature-comparator P values for superiority testing were calculated by two-sided unpaired Welch's unequal-variance t-tests on Log_{10} reduction summary statistics as described in Methods.*

Supplementary Table S4. Plasma log10 viral reduction from vehicle for each day (HCV Study).

Day	Sofosbuvir (clinical eq. dose)	MDL-001		
	Log_{10} reduction	Log_{10} reduction		
day 3	-0.0 ± 0.1	-0.1 ± 0.1		
day 6	1.0 ± 0.1	0.8 ± 0.1		
day 9	1.9 ± 0.1	1.7 ± 0.1		
day 12	2.4 ± 0.2	2.0 ± 0.1		
day 15	2.7 ± 0.2	2.3 ± 0.1		
day 18	2.7 ± 0.1	2.6 ± 0.1	**Superiority Comparison**	**Equivalence Comparison Difference 90% CI (MDL-001 - SOF)**
day 21	2.8 ± 0.1	2.9 ± 0.1	Non-Superior (*P* = 0.79)	Equivalent (0.0 to +0.2 log_{10})
day 24	3.0 ± 0.1	3.0 ± 0.1	Non-Superior (*P* = 0.61)	Equivalent (−0.1 to +0.1 log_{10})
day 28	3.3 ± 0.2	3.3 ± 0.1	Non-Superior (*P* = 0.75)	Equivalent (−0.2 to +0.1 log_{10})

Log_{10} reductions are shown as mean ± 95% CI. n = 6 per group. The MDL-001 versus sofosbuvir superiority comparisons were performed on animal-level plasma-viremia absolute reductions by two-sided unpaired Welch's unequal-variance t-tests (Day 21, P = 0.79; Day 24, P = 0.61; Day 28, P = 0.75). Equivalence was assessed by two one-sided tests (TOSTs) against a pre-specified margin of ±0.5 log10. See Methods.

Supplementary Table S5. Plasma HBsAg log10 reduction from vehicle for each day (HBV Study).

**Day**	**Oral TAF**	**Oral MDL-001**	
**Day 1**	0.0 ± 0.0	0.0 ± 0.0	
**Day 2**	0.1 ± 0.1	0.1 ± 0.0	
**Day 3**	0.2 ± 0.1	0.1 ± 0.0	
**Day 4**	0.3 ± 0.0	0.2 ± 0.0	
**Day 5**	0.3 ± 0.1	0.3 ± 0.0	
**Day 6**	0.4 ± 0.1	0.4 ± 0.0	
**Day 7**	0.5 ± 0.0	0.5 ± 0.0	**Superiority Comparison**
**Day 8**	0.6 ± 0.0	0.7 ± 0.0	MDL-001 Superior (*P* = 0.0042)
**Day 9**	0.7 ± 0.0	0.8 ± 0.0	MDL-001 Superior (*P* = 0.0005)
**Day 10**	0.8 ± 0.0	0.9 ± 0.0	MDL-001 Superior (*P* = 0.0003)
**Day 13**	1.0 ± 0.1	1.0 ± 0.0	MDL-001 Superior (*P* = 0.0037)
**Day 16**	1.1 ± 0.1	1.2 ± 0.0	MDL-001 Superior (*P* = 0.0005)
**Day 19**	1.2 ± 0.1	1.5 ± 0.1	MDL-001 Superior (*P* <0.0001)
**Day 21**	1.4 ± 0.2	1.7 ± 0.1	MDL-001 Superior (*P* = 0.0059)
**Day 24**	1.7 ± 0.3	1.9 ± 0.2	MDL-001 Non-superior (P = 0.0746)
**Day 27**	**1.9 ± 0.4**	**2.3 ± 0.3**	LLOQ reached in 3/6 of MDL-001 mice, 1/6 of TAF mice
**Day 28**	**2.4 ± 0.1**	**2.5 ± 0.0 (LLOQ)**	**LLOQ** reached in all MDL-001 mice, 5/6 of TAF mice

Log10 reductions are shown as mean ± 95% CI. n = 6 per group. The MDL-001 versus tenofovir alafenamide superiority comparisons were performed on log10-transformed per-animal values by two-sided unpaired Welch's unequal-variance t-tests. The assay lower limit of quantification was 1 ng/mL. Values at or below the limit were censored at the limit before transformation, so reductions on days when an arm reached the limit are lower bounds. See Methods.

Supplementary Table S6. Oral pharmacokinetics of MDL-001 in mouse plasma, lung, and liver after single oral doses of 25, 50, or 75 mg/kg in suspension. *Tissues were sampled at 1, 2, 4, 8, 16, and 24 h. Units are ng/mL for plasma and ng/g for tissues. Parameters are* t_{max}, C_{max}, C_{24h}, $AUC_{0–24h}$, *and* $t_{1/2}$. *NC indicates not calculated.*

**Oral PK of MDL-001 in Mouse Plasma**				
**Parameter**	**Units**	**Dose (mg/kg)**		
		25	50	75
**T_{max}**	**h**	2	1	1
**C_{max}**	**ng/g**	122	155	280
**C_{24h}**	**ng/g**	1.6	7.2	20.1
**AUC_{0-24h}**	**ng-h/g**	670	937	2227
**$T_{1/2}$**	**h**	< 8	NC	8
**Oral PK of MDL-001 in Mouse Lung**				
**Parameter**	**Units**	**Dose (mg/kg)**		
		25	50	75
**T_{max}**	**h**	2	4	4
**C_{max}**	**ng/g**	4722	8136	13348
**C_{24h}**	**ng/g**	133	420	2310
**AUC_{0-24h}**	**ng-h/g**	25729	67778	168258
**$T_{1/2}$**	**h**	9	NC	8
**Oral PK of MDL-001 in Mouse Liver**				
**Parameter**		**Dose (mg/kg)**		
		**25**	**50**	**75**
**T_{max}**	**h**	2	1	4
**C_{max}**	**ng/g**	8636	16060	19870
**C_{24h}**	**ng/g**	184	472	2414
**AUC_{0-24h}**	**ng-h/g**	43746	85461	186764
**$T_{1/2}$**	**h**	8	9	9
**Lung Partition**		**Dose (mg/kg)**		
		**25**	**50**	**75**
**C_{max}-derived**	**K_p**	39	52	48
**AUC_{0-24h}-derived**	**K_p**	38	72	76
**Liver Partition**		**Dose (mg/kg)**		
		**25**	**50**	**75**
**C_{max}-derived**	**K_p**	71	104	71
**AUC_{0-24h}-derived**	**K_p**	65	91	84

Supplementary Table S7. Rat plasma pharmacokinetics of Oral MDL-001 formulation. *Non-compartmental pharmacokinetic parameters in male Sprague-Dawley rats following a single oral dose at 400 mg/kg (n = 3). Plasma was sampled at 0.5, 1, 2, 4, 6, 10, and 24 h post-dose.*

Rat Plasma PK of MDL-001 (Optimized Oral Solution Formulation)		
Parameter	***Units***	***Dose (mg/kg)***
		400
Actual delivered dose	***mg/kg***	*543 ± 12.1*
t_{max} (range)	***h***	*4 (2–10)*
C_{max}	***ng/mL***	*641 ± 18.5*
C_{24h}	***ng/mL***	*72.8 ± 67.9*
$t_{1/2}$	***h***	*NC*
AUC_{last}	***h·ng/mL***	*7150 ± 1470*
AUC_{∞}	***h·ng/mL***	*NC*

Supplementary Table S8. Cross-Species In Vitro Intrinsic Clearance of MDL-001.

**Species**	**$t_{½}$ (min)**	**$CL_{int,u}$ (µL/min/million)**	**In Vivo CL (mL/min/kg)**	**Hepatic Extraction Ratio (%)**	**Clearance Classification**
Mouse	17.8	458	27.3	23	Low
Rat	18.5	440	22.2	33	Intermediate
Dog	16.9	484	7.97	26	Low
Monkey	22.4	364	2.13	5	Low
Human	53.2	153	1.68	8	Low

Supplementary Table S9. Metabolite profile for MDL-001 across species in cryopreserved hepatocytes. *Human metabolites greater than 1.5% fraction are bold.*

Metabolite	Identification	Human (%)	Mouse (%)	Rat (%)	Dog (%)	Monkey (%)	Buffer (%)
**MDL-001**	**Parent compound**	**33.7**	**6.6**	**31.9**	**8.4**	**7.5**	**95.5**
**M1**	**Oxidation in piperidine (N-oxide)**	**2.5**	**0.2**	**12.2**	**3.3**	**1.0**	**4.4**
M7	Oxidative dealkylation + ox + dehydrogenation	1.3	0.8	2.0	2.2	2.9	–
**M10**	**Glucuronide conjugation (phenol)**	**45.0**	**31.1**	**8.0**	**12.5**	**72.1**	**–**
**M11**	**Glucuronide conjugation (phenol)**	**5.0**	**45.4**	**16.8**	**48.7**	**6.7**	**–**
M16	Ox in piperidine N-oxide + glucuronide (phenol)	0.9	0.6	1.4	0.8	1.4	–
**M30**	**Sulfo-conjugation**	**7.9**	**0.1**	**3.4**	**3.1**	**0.9**	**–**
M31	Ox in piperidine N-oxide + sulfo	0.9	–	4.8	5.4	0.2	–

Supplementary Table S10. Calculation of hERG safety margin from in vitro IC_{50} and projected unbound human plasma C_{max} at the minimally efficacious dose.

Parameter	Value
**hERG IC_{50} (Predictor fluorescence-polarization assay; no plasma protein in incubation)**	13 µM (13,000 nM)
**Rat plasma C_{max} at a minimally efficacious dose**	230 ng/mL
**Human plasma free fraction (F_u)**	0.005
**Projected unbound human plasma C_{max} (= $C_{max} \times F_u$; converted to nM using MW 456.58 g/mol)**	1.15 ng/mL = 2.5 nM
**hERG IC_{50}/ unbound C_{max} safety margin**	>5,200-fold

Supplementary Table S11. Summary of duration, animal, and route of administration for the tolerability studies of MDL-001 in mice and rats (totaling 376 animals across seventeen experiments).

Study Duration	N (animals)	Route
3 days	6 mice	Oral
3–4 days	22 mice	Oral
3 days	36 mice	Oral
10 days	30 mice	Oral
10 days	18 mice	Oral
28 days	18 mice	Oral
10–16 days	24 mice	Oral
28 days	18 mice	Oral
4–6 days	18 mice	Oral
4–7 days	6 mice	Oral
7 days	3 mice	Oral
1 dose	90 mice	Oral
5 days	48 mice	Oral
1 dose	12 rats	Oral
1 dose	9 rats	Oral
1 dose	6 rats	IV
1 dose	12 rats	Oral
